# Severe constipation‐associated postprandial abdominal pain in a pregnant native‐liver biliary atresia survivor after Kasai portoenterostomy: A case report

**DOI:** 10.1002/jpr3.70253

**Published:** 2026-10-14

**Authors:** Akram Alnounou, Ajay Katwala, Mohammad Alabbas, Bisher Sawaf, Arghyadeep Ganguly, EzzElDien A. Ibrahim, Fnu Vikash

**Affiliations:** ^1^ Department of Internal Medicine Western Michigan University Homer Stryker MD School of Medicine Kalamazoo Michigan USA; ^2^ College of Health Sciences Marquette University Milwaukee Wisconsin USA; ^3^ Department of Gastroenterology, Hepatology, and Nutrition Cleveland Clinic Cleveland Ohio USA; ^4^ Department of Internal Medicine University of Toledo Medical Center Toledo Ohio USA; ^5^ Department of Medicine University of Missouri‐Columbia Columbia Missouri USA

**Keywords:** chronic liver disease, fecal impaction, peripartum care, portal hypertension

## Abstract

An 18‐year‐old woman with congenital biliary atresia status post Kasai portoenterostomy and magnetic resonance imaging‐reported chronic liver disease presented at 32 weeks’ gestation with 6 weeks of worsening postprandial epigastric/right upper quadrant pain, nausea, and poor oral intake. Evaluation showed preserved hepatic synthetic function and no demonstrated clinically significant portal hypertension. Endoscopy excluded esophageal and gastric varices, computed tomography showed no imaging evidence of pancreatitis, biliary obstruction, or other acute intra‐abdominal pathology, computed tomography angiography excluded mesenteric ischemia, and gastric emptying scintigraphy excluded gastroparesis. Nonreassuring fetal surveillance prompted preterm induction at approximately 33 weeks 4 days. Postpartum symptoms persisted, and imaging review identified severe constipation with rectal impaction/fecalization as the dominant treatable contributor. This case broadens the peripartum phenotype reported in native‐liver biliary atresia survivors by emphasizing constipation‐associated postprandial abdominal pain, poor oral intake, nutritional risk, and medication‐related bowel dysfunction rather than variceal bleeding or hepatic decompensation alone.

## Graphical abstract

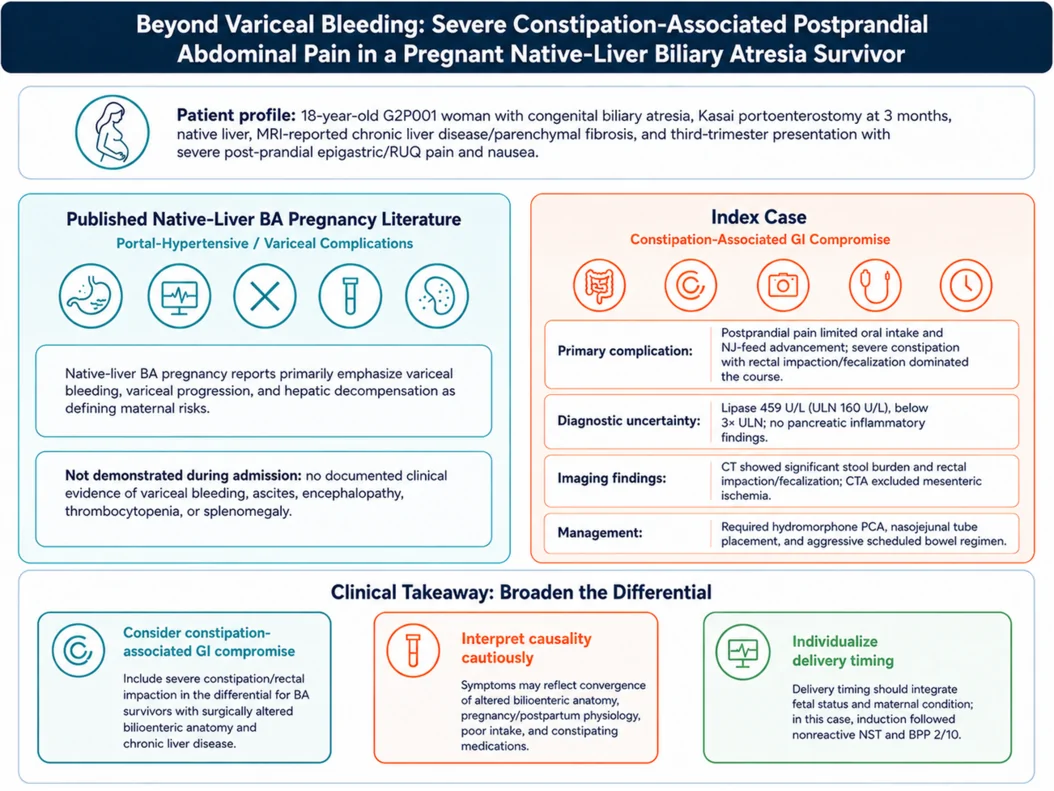

## 1 INTRODUCTION

Biliary atresia survivors who retain their native liver after Kasai portoenterostomy are uncommon in adulthood and remain at risk for chronic liver disease, cholangitis, portal hypertension, varices, gastrointestinal bleeding, and eventual liver transplantation.^1^, ^2^, ^3^ Pregnancy in this subgroup is increasingly encountered but remains poorly characterized. Published native‐liver biliary atresia pregnancy reports have largely emphasized variceal progression, variceal hemorrhage, and hepatic decompensation.^1^, ^4^, ^5^, ^6^ This framing is clinically important, but it can also narrow diagnostic attention when a pregnant biliary atresia‐Kasai survivor presents with abdominal pain and poor oral intake without demonstrated portal‐hypertensive complications.

We report an 18‐year‐old pregnant native‐liver biliary atresia survivor whose peripartum course was dominated by severe constipation with rectal impaction/fecalization, refractory postprandial abdominal pain and poor oral intake, rather than variceal hemorrhage or hepatic decompensation. The aim is not to establish a biliary atresia‐specific causal mechanism, but to highlight an underrecognized, treatable gastrointestinal and nutritional challenge in this high‐risk population. A visual summary of the clinical presentation, diagnostic findings, and key teaching points is provided in the Figure S1.

## 2 CASE REPORT

An 18‐year‐old gravida 2 para 0‐0‐1‐0 woman at 32 weeks’ gestation had congenital biliary atresia treated with Kasai portoenterostomy at 3 months of age and cholecystectomy in childhood. Six weeks before admission, abdominal magnetic resonance imaging obtained for persistent postprandial pain and nausea reported cirrhotic morphology/parenchymal fibrosis and surgical absence of the gallbladder; magnetic resonance elastography and dedicated vascular sequences were not performed. Her pre‐pregnancy history included one episode of ascending cholangitis at age 16 treated with intravenous antibiotics. She had no documented pre‐pregnancy hepatology evaluation, elastography, variceal screening, portal venous patency assessment, hepatic venous pressure gradient measurement, or baseline Child‐Pugh or Model for End‐Stage Liver Disease‐Sodium score.

She presented with at least 6 weeks of worsening postprandial epigastric/right upper quadrant pain, nausea, subjective fever, and poor oral intake. She was afebrile and hemodynamically stable. Admission laboratory testing showed white blood cell count 8.9 K/μL, platelets 295 K/μL, international normalized ratio 1.0, albumin 3.5 g/dL, total bilirubin 0.5 mg/dL, aspartate aminotransferase 24 IU/L, alanine aminotransferase 19 IU/L, alkaline phosphatase 173 IU/L, and lipase 42 U/L. Examination showed no ascites or encephalopathy. A calculated Child‐Pugh score of 6 and Model for End‐Stage Liver Disease‐Sodium score of 6 were used only for risk stratification, not as proof of cirrhosis. There was no clinical evidence of acute cholangitis, and antibiotics were not started. Right upper quadrant ultrasound showed mild right hydronephrosis, postoperative absence of the gallbladder, and limited pancreatic visualization.

On hospital day 8, fetal surveillance became nonreassuring with a nonreactive non‐stress test and biophysical profile 2/10 at approximately 33 weeks 3 days. Estimated fetal weight was 1907 g, with normal amniotic fluid. Because of nonreassuring fetal testing in the setting of ongoing severe maternal symptoms, induction was performed on hospital day 9 at approximately 33 weeks 4 days, followed by uncomplicated vaginal delivery. The neonate required neonatal intensive care for approximately 1 month.

Postpartum, severe postprandial epigastric/right upper quadrant pain and nausea continued to limit oral intake despite acid suppression, antiemetics, ursodiol, and opioid analgesia. Computed tomography of the abdomen and pelvis on hospital day 9 showed no acute intra‐abdominal process, no computed tomography evidence of pancreatitis, mild intrahepatic biliary dilation consistent with post‐Kasai anatomy, and significant stool burden. Lipase later peaked at 459 U/L on hospital day 21 (institutional upper limit of normal, 160 U/L). Upper endoscopy on hospital day 11 showed a normal esophagus, stomach, and duodenum without esophageal or gastric varices. A nasojejunal tube was placed on hospital day 17 for nutritional support; feeds reached 40 mL/hour but could not be advanced to goal because higher rates worsened the same abdominal pain, and the tube occluded on hospital day 29. No vomiting or abdominal distension was documented with oral or nasojejunal feeding. Gastric emptying scintigraphy was normal, and computed tomography angiography on hospital day 28 showed no mesenteric ischemia or pancreatic inflammatory findings.

Imaging review emphasized marked constipation with rectal impaction/fecalization (Figure 1). Pregnancy/postpartum physiology and medication exposures, particularly opioids and ondansetron, were considered important contributors. Ondansetron was minimized when feasible, and a scheduled bowel regimen with polyethylene glycol, psyllium, and lactulose was used. No ascites, encephalopathy, splenomegaly, thrombocytopenia, varices, or variceal bleeding was documented. She was transferred to a tertiary center on hospital day 33; hepatology found no hepatic decompensation or urgent transplant indication, and luminal gastroenterology recommended removal of enteric access, oral intake as tolerated, and outpatient follow‐up. She was discharged after a 3‐day tertiary‐center stay. The clinical timeline of the hospital course is summarized in Figure 2.

**Figure 1:**
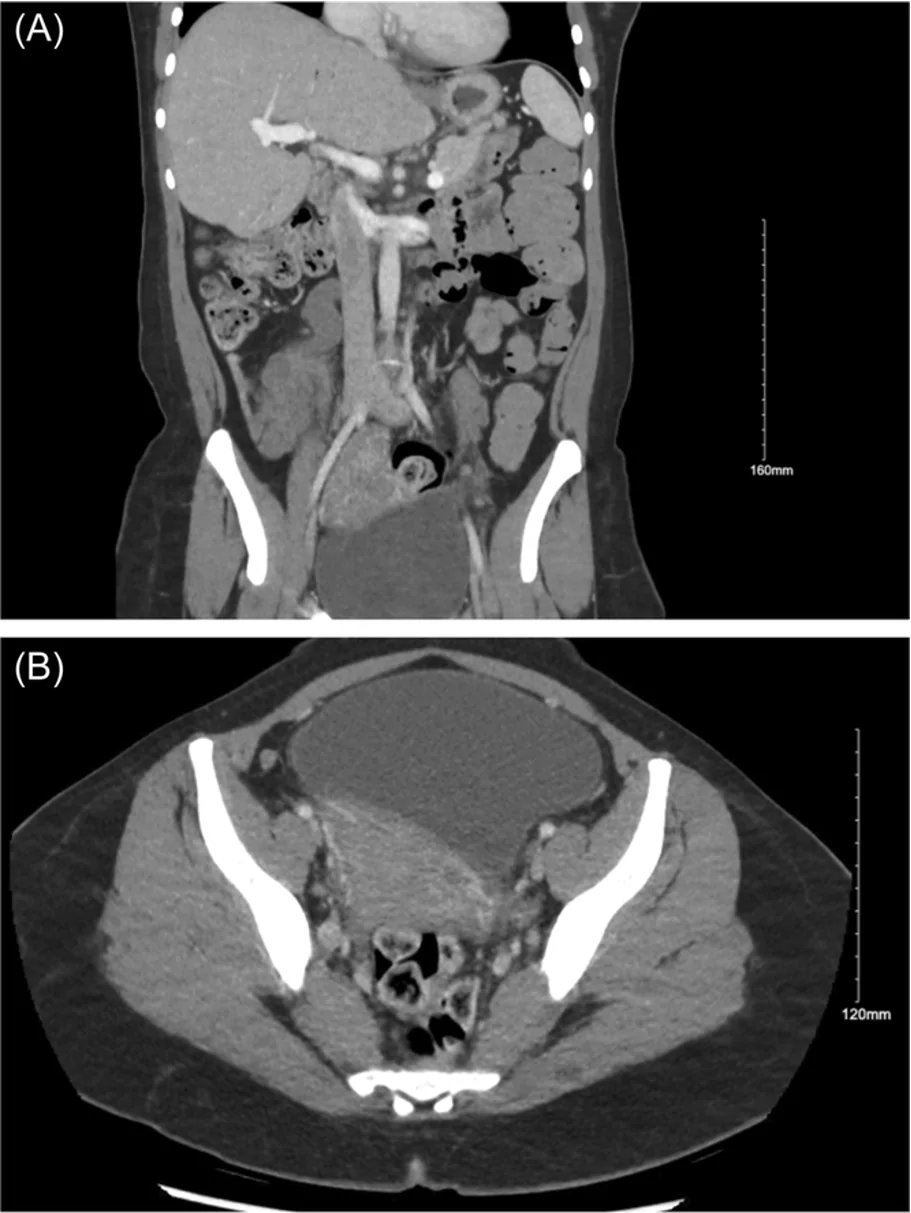
Computed tomography demonstrating severe fecal loading and distal fecal impaction. (A) Coronal contrast‐enhanced computed tomography of the abdomen and pelvis demonstrates diffuse colonic fecal loading with interspersed gas, consistent with severe stool burden. (B) Axial contrast‐enhanced computed tomography of the pelvis demonstrates dense rectal fecal material consistent with distal fecal impaction. Mild intrahepatic biliary dilation, consistent with post‐Kasai portoenterostomy anatomy, was also noted but is not shown. These findings supported initiation of an aggressive bowel regimen in the setting of prolonged postpartum abdominal pain and poor oral intake, pregnancy/postpartum physiology, and constipating medication exposure.

**Figure 2:**
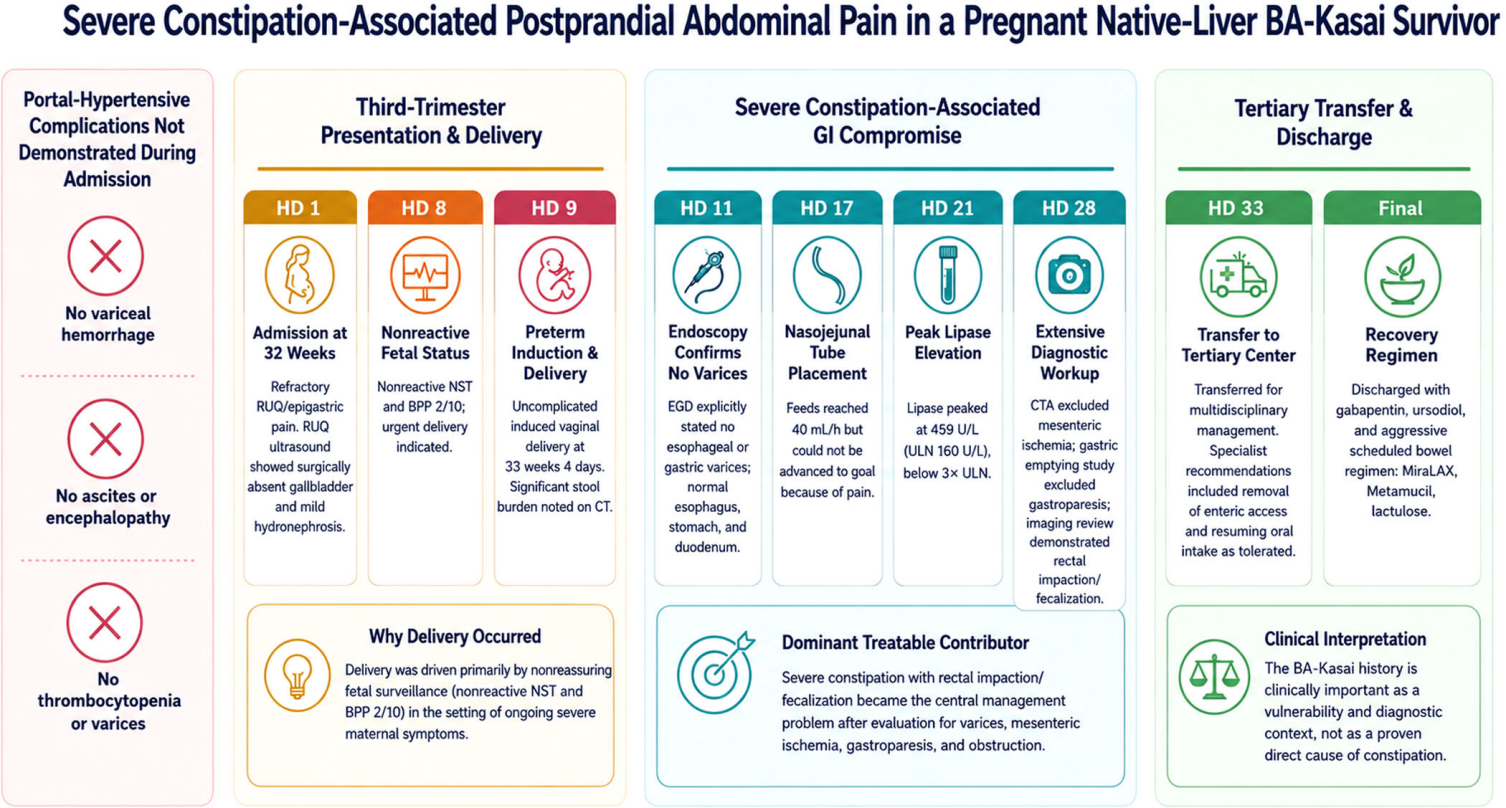
Clinical timeline of the hospital course. Key clinical events, diagnostic evaluations, interventions, and outcomes are organized by hospital day. The timeline highlights the absence of demonstrated portal‐hypertensive complications during admission, nonreassuring fetal surveillance prompting preterm induction, exclusion of varices and other structural etiologies, and severe constipation with rectal impaction/fecalization as the dominant treatable contributor to abdominal pain and impaired nutritional intake.

## 3 DISCUSSION

Pregnancy in cirrhosis is associated with increased maternal and perinatal risk, including preterm delivery and low birth weight.^7^ In biliary atresia survivors retaining native liver, published pregnancies have been dominated by variceal complications.^1^, ^4^, ^5^, ^6^ Our case is notable because it adds a different peripartum phenotype: severe constipation‐associated postprandial abdominal pain, poor oral intake, and pain‐limited advancement of enteral feeds, while variceal bleeding, clinically significant portal hypertension, biliary obstruction, mesenteric ischemia, gastroparesis, and hepatic decompensation were not demonstrated.

Acute pancreatitis was considered because lipase rose from 42 U/L on admission to 459 U/L on hospital day 21. NASPGHAN/INSPPIRE criteria require at least two of three findings: compatible abdominal pain, serum amylase or lipase ≥3 times the upper limit of normal, or characteristic imaging findings.^8^ Although epigastric pain was present, it predated the lipase elevation by weeks and was unchanged at the peak; lipase remained below three times the upper limit of normal (upper limit of normal, 160 U/L), and cross‐sectional imaging showed no pancreatic inflammatory findings. Thus, diagnostic criteria for acute pancreatitis were not met, and pancreatitis was not considered the primary explanation for the prolonged clinical course.

The main teaching point is diagnostic discipline. In a biliary atresia‐Kasai survivor, clinicians should screen for varices and hepatic decompensation, but should not attribute all abdominal pain or poor oral intake to portal hypertension or biliary disease. Standard magnetic resonance imaging morphology alone can overstate or incompletely characterize compensated chronic liver disease; elastography and vascular assessment provide more direct risk information when available.^9^, ^10^ In this patient, normal platelets, preserved synthetic function, absence of ascites or encephalopathy, and negative endoscopy argued against clinically significant portal hypertension during admission, although her baseline hepatic phenotype remained incompletely characterized.

The second teaching point is that constipation can become a major postpartum nutritional threat. Pregnancy can slow bowel transit, and opioids and ondansetron may worsen constipation.^11^ In this case, stool burden with rectal impaction/fecalization was the dominant treatable abnormality after exclusion of structural, ischemic, gastroparetic, variceal, and obstructive etiologies. The association should be presented cautiously: constipation‐associated functional gastrointestinal compromise is a clinical inference, not proof of a biliary atresia‐specific mechanism. This distinction preserves the paper's novelty while avoiding a causal overclaim; the publishable message is clinical recognition and management, not discovery of a new pathophysiologic syndrome.

## 4 CONCLUSION

This case should not be interpreted as supporting preterm delivery for gastrointestinal symptoms alone. Delivery was prompted by nonreassuring fetal surveillance in the context of ongoing maternal illness. Limitations include the absence of outpatient follow‐up, incomplete pre‐pregnancy hepatic staging, no spleen‐size or dedicated portal vascular assessment, and no formal small‐bowel or colonic motility testing. Even with these limitations, the case expands the reported risk spectrum in native‐liver biliary atresia pregnancy and supports multidisciplinary postpartum follow‐up focused not only on variceal surveillance, but also nutrition, medication‐related constipation risk, bowel function, and hepatic reserve.

## CONFLICT OF INTEREST STATEMENT

The authors declare no conflicts of interest.

## ETHICS STATEMENT

Ethical approval was not required for this single case report because it did not meet the U.S. federal regulatory definition of human‐subjects research under 45 Code of Federal Regulations 46 and was not considered human‐subjects research under institutional policy. Written informed consent was obtained from the patient for publication of the details of this medical case and any accompanying images.

## Supporting information


**Supplementary Figure. Visual abstract: Beyond Variceal Bleeding: Severe Constipation‐Associated Postprandial Abdominal Pain in a Pregnant Native‐Liver Biliary Atresia Survivor**. This visual abstract summarizes the clinical presentation, diagnostic findings, and key clinical takeaways of severe constipation‐associated gastrointestinal compromise, postprandial abdominal pain, and poor oral intake in an 18‐year‐old gravida 2 para 0‐0‐1‐0 woman with biliary atresia, native liver, and Kasai portoenterostomy at 3 months of age who presented at 32 weeks’ gestation.

## Data Availability

Data sharing is not applicable to this article as no datasets were generated or analyzed during the current study.
